# Supplementary material for: Mapping of QTLs Associated with Biological Nitrogen Fixation Traits in Peanuts (Arachis hypogaea L.) Using an Interspecific Population Derived from the Cross between the Cultivated Species and Its Wild Ancestors
Source: Genes (Basel). 2023 Mar 26;14(4):797. doi: 10.3390/genes14040797 (PMC10138160; doi:10.3390/genes14040797)
Supplement: Supplementary file 1 [file genes-14-00797-s001.zip › genes-2263897-supplementary.pdf]

Supplementary Materials

Table S1. Summary of significant line × trait associations with their QTL effects identified in all treatments in 2017 environment

CSSL	Chromosome	SPAD			TB		
		QTL effect (%)			QTL effect (%)		
		-N	+N	-N+ISRA400	-N	+N	-N+ISRA400
12CS_023	A01	ns	ns	ns	ns	ns	-48.76**
12CS_096	A01	ns	ns	ns	ns	ns	-61.13***
12CS_071	A01	ns	ns	ns	ns	ns	-41.65*
12CS_016	A01, **B10**	ns	ns	ns	ns	ns	-58.32**
12CS_041	A02	ns	ns	ns	-63.18*	ns	ns
12CS_051	A02, **A08**, **B02**	ns	ns	-65.29***	ns	ns	-71.51***
12CS_052	A02, **B11**	ns	ns	ns	ns	ns	-62.55***
12CS_004	A03	ns	ns	-29.23***	-64.24*	ns	-71.95***
12CS_055	A03	ns	ns	ns	-64.46*	ns	ns
12CS_042	A03	ns	ns	ns	-69.46*	ns	-41.04*
12CS_027	A04, **A07**, **B01**, **B02**	ns	ns	-24.61*	ns	ns	-47.38**
12CS_098	A04	ns	ns	ns	-65.18*	ns	-48.53*
12CS_090	A04, **A09**	ns	ns	ns	ns	ns	-45.74*
12CS_022	A05	ns	ns	ns	ns	ns	-47.87*
12CS_039	A08, **B11**	ns	ns	ns	ns	ns	-39.73*
12CS_084	A08, **A01**	ns	ns	-33.79***	ns	ns	-53.28**
12CS_028	A09, **B10**	ns	ns	ns	ns	ns	-49.2*
12CS_106	B01	ns	ns	ns	-61.4**	ns	ns
12CS_044	B02	ns	ns	-47.89***	ns	ns	-66.09***
12CS_118	B03, **A06**, **A09**	ns	ns	ns	ns	ns	-52.02*
12CS_059	B04	ns	ns	ns	ns	ns	-49.12**
12CS_063	B06, **B10**	ns	ns	ns	ns	ns	-44.63**
12CS_076	B06	ns	ns	ns	ns	ns	-52.02**
12CS_060	B06, **B08**, **A07**	ns	ns	-41.77***	ns	ns	-69.63***
12CS_047	B06	ns	ns	ns	ns	ns	-57.13***
12CS_081	B07	ns	ns	ns	ns	ns	-60.27**
12CS_048	B07	ns	ns	ns	ns	ns	-41.04*
12CS_006	B08, **A01**, **B03**	ns	ns	ns	ns	ns	-50.57*
12CS_064	B10	ns	ns	ns	ns	ns	-47.1**

The values for SPAD (leaf chlorophyll content) and TB (total biomass) represent the relative difference (QTL effect) between the CSSLs and Fleur11. -N and +N treatments are respectively negative (without nitrogen) and positive (with urea) controls. -N+ISRA400 treatment: without nitrogen + inoculation with

Bradyrhizobium vignae Strain ISRA400. Chromosome indicates the linkage group which carries the target wild segment. Chromosomes mentioned in bold constitute supplementary wild segments that were not the initial targeted during the development of the CSSL population. *, ** and ***: significant at $p < 0.05$, $p < 0.01$ and $p < 0.001$ respectively, ns: not significant.

Table S2. Summary of significant line × trait associations with their QTL effects identified in all treatments in 2018 environment

CSSL	Chromosome	SPAD			TB		
		QTL effect (%)			QTL effect (%)		
		-N	+N	-N+ISRA400	-N	+N	-N+ISRA400
12CS_051	A02, **A08**, **B02**	ns	ns	-43.26***	ns	ns	-56.43*
12CS_004	A03	ns	ns	-64.77***	-52.79**	ns	-64.68**
12CS_072	A03	ns	ns	ns	-47.01**	-37.21*	ns
12CS_103	A03, **A08**	ns	ns	-26.76***	ns	ns	-60.58**
12CS_055	A03	ns	ns	ns	-47.11*	ns	ns
12CS_042	A03	ns	ns	ns	-49.57*	ns	ns
12CS_098	A04	ns	ns	ns	ns	ns	-55.10*
12CS_108	A04, **B01**	ns	ns	-22.79***	-52.34**	ns	ns
12CS_086	A04	ns	ns	ns	-53.54***	ns	ns
12CS_022	A05	ns	ns	ns	-57.96***	ns	ns
12CS_121	A06, **A10**, **B03**, **B08**	ns	ns	ns	-38.63*	ns	ns
12CS_092	A07	ns	ns	ns	-48.64**	ns	ns
12CS_062	A07, **B10**	ns	ns	ns	ns	ns	-26.32***
12CS_034	A07, **A03**	ns	ns	ns	-48.37*	ns	-52.76*
12CS_084	A08, **A01**	ns	ns	-61.67***	ns	ns	-58.35*
12CS_028	A09, **B10**	ns	ns	-15.17**	ns	ns	ns
12CS_031	A09, **B10**	ns	ns	ns	-41.45**	ns	ns
12CS_018	A09	ns	ns	ns	-46.44*	ns	ns
12CS_078	A10, **B03**	ns	ns	ns	-58.57***	ns	ns
12CS_106	B01	ns	ns	ns	-49.52*	ns	ns
12CS_119	B02, **A01**	ns	ns	ns	-49.9**	ns	ns
12CS_044	B02	ns	ns	-73.49***	ns	ns	-55.36**
12CS_118	B03, **A06**, **A09**	ns	ns	ns	-57.93*	ns	-53.04*
12CS_001	B03, **B01**	ns	ns	ns	-45.65*	ns	ns
12CS_059	B04	ns	ns	ns	-48.5*	ns	ns

12CS_101	B05, **A08**	ns	ns	ns	-55.69**	ns	ns
12CS_081	B07	ns	ns	ns	-41.91*	ns	-57.06*
12CS_079	B09, **A10**, **B03**	ns	ns	ns	-37.94*	ns	ns
12CS_064	B10	ns	ns	ns	-41.47*	ns	ns
12CS_066	B10	ns	ns	ns	-47.23*	ns	ns
12CS_010	B11	ns	0.1*	ns	ns	ns	ns

The values for SPAD (leaf chlorophyll content) and TB (total biomass) represent the relative difference (QTL effect) between the CSSLs and Fleur11. -N and +N treatments are respectively negative (without nitrogen) and positive (with urea) controls. -N+ISRA400 treatment: without nitrogen + inoculation with *Bradyrhizobium vignae* Strain ISRA400. Chromosome indicates the linkage group which carries the target wild segment. Chromosomes mentioned in bold constitute supplementary wild segments that were not the initial targeted during the development of the CSSL population. *, ** and ***: significant at $p < 0.05$, $p < 0.01$ and $p < 0.001$ respectively, ns: not significant.

Table S3. Characteristics of the QTLs associated to leaf chlorophyll content and total biomass in inoculated treatment

**Trait**	**LG**	**2017**			**2018**		
		**Closest markers**		**Conf. Int (cM)**	**Closest markers**		**Conf. Int (cM)**
SPAD	A02	Seq12E03_A	RM2H10_A	43.85-55.35	Seq12E03_A	RM2H10_A	43.85-55.35
	A03	TC4G02_A	Ah-30_A	0-16.6	TC4G02_A	Ah-30_A	0-16.6
	A03	.	.	.	gi-4925_A	PM238_A	57.2-74.2
	A04	RN12E01_A	TC9E08_A2	0-48.975	.	.	.
	A04				TC9E08_A2	TC9E08_A1	48.975-90.975
	A07	Ah3TC23E04_A	PM042_A1	12.1-28.525	.	.	.
	A07	.	.	.	PM042_A1	TC9H08_A	28.525-60.0
	A08	.	.	.	RM5G08_A	Ad90F2_A	46.15-75.525
	A08	Ad90F2_A	TC1E05_A	75.525-85.375	Ad90F2_A	TC1E05_A	75.525-85.375
	A09	.	.	.	TC9B07_A	seq4G02_A	7.125-49.375
	B01	.	.	.	Ah-3_B	Seq19H03_B2	66.725-87.525
	B02	Seq1B09_B	Ah3TC13E05_B	24.55-43.25	Seq1B09_B	Ah3TC13E05_B	24.55-43.25
	B06	TC3H07_B	Ah3TC19F05_B	16.275-50.95	.	.	.
	B08	Ad90F2_B	IPAHM229_B	5.25-21.25	.	.	.
	B10	AC2B03_B	AC2B03_B	48.025-55.525	.	.	.
TB	A01	TC2E05_A	IPAHM287_A	0-9.675	.	.	.

A01	seq16G08_A	seq16G08_A	42.325-47.7	.	.	.
A01	Seq4A06_A	TC3H02_A	111.125-119.975	.	.	.
A02	RM2H10_A	RM2H10_A	55.35-63.125	.	.	.
A02	.	.	.	Seq12E03_A	RM2H10_A	43.85-55.35
A03	.	.	.	gi-4925_A	PM238_A	57.2-74.2
A04	TC9E08_A1	TC11B04_A1	90.975-113.15			
A04	.	.	.	Seq18A08_A3	TC9E08_A1	57.625-90.975
A05	gi-0620_A	Ah-614_A	0-46.8	.	.	.
A06	TC11A04_A	TC11A04_A	29.825-43.925	.	.	.
A08	.	.	.	RM5G08_A	Ad90F2_A	46.15-75.525
A08	Ad90F2_A	TC1E05_A	75.525-85.375	Ad90F2_A	TC1E05_A	75.525-85.375
A09	TC9B07_A	seq4G02_A	7.125-49.375			
A09	RN35H04_A	gi-1107_A	72.1-96.1	.	.	.
B02	Seq1B09_B	Ah3TC13E05_B	24.55-43.25	Seq1B09_B	Ah3TC13E05_B	24.55-43.25
B03	TC7E04_B	IPAHM093_B	1.725-22.575	.	.	.
B04	IPAHM108_B	Ah3TC12A01_B	40.875-57.225	.	.	.
B06	PM137_B	Ah3TC19F05_B	31.3-50.95	.	.	.
B07	seq2E06_B	seq2E06_B	0-2.05	.	.	.
B07	Seq5D05_B	Seq5D05_B	14.5-21.275	.	.	.
B08	Ah3TC31E08_B	Ah3TC31E08_B	26.95-29.8	.	.	.
B10	Ah3TC22G05_B	Ah3TC22G05_B	0-8.5	.	.	.
B10	AC2B03_B	AC2B03_B	48.025-55.525	.	.	.
B11	TC3E02_B	Ah3TC23E04_B	0-15.1	.	.	.

SPAD: leaf chlorophyll content; TB: total biomass. LG indicates the linkage group which carries the wild segment responsible for the phenotype. Conf. Int represents the confidence interval of the QTL on the chromosome.

Table S4. Orthologs of nodulation genes located on the regions containing the QTLs

CSSLs	Phenotype description	QTL position	Candidate genes			
		Chromosome (start-end pb)	Gene name	Ortholog genes	Description	Symbiotic functional role
12CS_004	Decreases SPAD, TB, ARA, NDW, NN, and NDW/NN	A03: 121100027 - 127945426	*Aradu.51WYZ*	*MtGS1-2*	Plastid localized enzyme essential in nitrogen metabolism nodules	Not defined
			Aradu.WG73C	*LjSIE3*	Enzyme (Ligase, CTLH/CRA C-terminal to LisH motif domain). Downregulation of *LjSIE3* inhibits infection thread development and nodule organogenesis.	Early Signaling
			Aradu.P8MSW	*LjSYMRK*	Enzyme (Kinase; Leucine rich repeat N-terminal domain). *LJSYMRK* mutants are unable to form root nodules and arbuscular mycorrhiza. They exhibit root hairs deformation, but no infection thread formation.	Early Signaling
			Aradu.J0SGA	*LjEPR3*	Enzyme (Protein kinase domain) controlling the rhizobial infection. *LjEPR3* plant mutants are defective in perception of bacterial exopolysaccharides.	Early Signaling

			Aradu.L8SVN	*MtNAC969*	Transcription factor (NAC domain). RNAi of *MtNAC969* induces the accumulation of amyloplasts in the nitrogen-fixing zone and leads to nodule senescence	Senescence
			Aradu.JZT13	*GmINS1*	Cell wall component (Trehalose-phosphate synthase). Overexpression of *GmINS1* increases nodule number and infection cell abundance.	Nodule organogenesis
			Aradu.UYZ93	*MtCRE1*	Enzyme (Histidine kinase, Bacterial sensor protein C-terminal signature). RNAi of *MtCRE1* increases number of lateral roots and strongly reduces nodulation	Nodule organogenesis
12CS_044	Decreases SPAD, TB, ARA, and NDW/NN	B02: 41413540 - 77881439	*Araip.QVW26*	*GmPT5/7*	Phosphate transporter. Overexpression or knockdown Of *GmPT5* in soybean mutants affect nodulation and plant growth.	Nodule functioning
			Araip.5U4XM	*MtVAMP721d/e*	Membrane protein (Vesicle trafficking). Silencing of MtVAMP had a minor effect in nodulation, but blocks the formation of symbiosome as well as	Symbiosome formation

					arbuscule formation in mycorrhizal symbiosis	
			Araip.RJS33	*MtNOOT*	Transcription factor (BLADE-ON-PETIOLE family). *M. truncatula* mutants exhibit one or multiple toots in an apical position on the fully developed nodule	Nodule organogenesis
			Araip.X1RXQ	*LjFEN1*	Enzyme (Homocitrate Synthase). *L. japonicus* mutants form morphologically normal but, ineffective nodules	Bacterial maturation
12CS_051	Decreases SPAD, TB, and NDW, and induces the collapse of nodule tissue	A02: 30278634 - 66574828	*Aradu.ZC0SW*	*GmPT5/7*	Phosphate transporter. Overexpression or knockdown Of *GmPT5* in soybean mutants affect nodulation and plant growth.	Nodule functioning
			Aradu.Q6WYU	*MtVAMP721d/e*	Membrane protein (Vesicle trafficking). Silencing of MtVAMP had a minor effect in nodulation, but blocks the formation of symbiosome as well as arbuscule formation in mycorrhizal symbiosis	Symbiosome formation
			Aradu.31ZLJ	*MtNOOT*	Transcription factor (BLADE-ON-PETIOLE family). *M. truncatula* mutants	Nodule organogenesis

					exhibit one or multiple toots in an apical position on the fully developed nodule	
			Aradu.F9JCM	*LjFEN1*	Enzyme (Homocitrate Synthase). *L. japonicus* mutants form morphologically normal but, ineffective nodules	Bacterial maturation
12CS_084	Decreases SPAD and TB	A08: 35192648 - 40925213	*Aradu.GZF8P*	*MtPNO1*	RNA-binding pno1-like protein. *M. truncatula* mutants produce approximately 60% of the number of nodules on wild-type plants.	Nodule organogenesis
			Aradu.3LM76	*LjMATE1*	Transporter (Citrate). LjMATE1 knockdown line produces small nodule than the wild plant, and low nitrogenase activity as well as the expression level of leghemoglobin.	Nodule functioning

SPAD: leaf chlorophyll content, TB: total biomass, NDW: nodule dry weight, NN: nodule number, ARA: acetylene reduction assay. Information regarding the description and symbiotic function of these ortholog genes are well reviewed by Roy et al. [29].

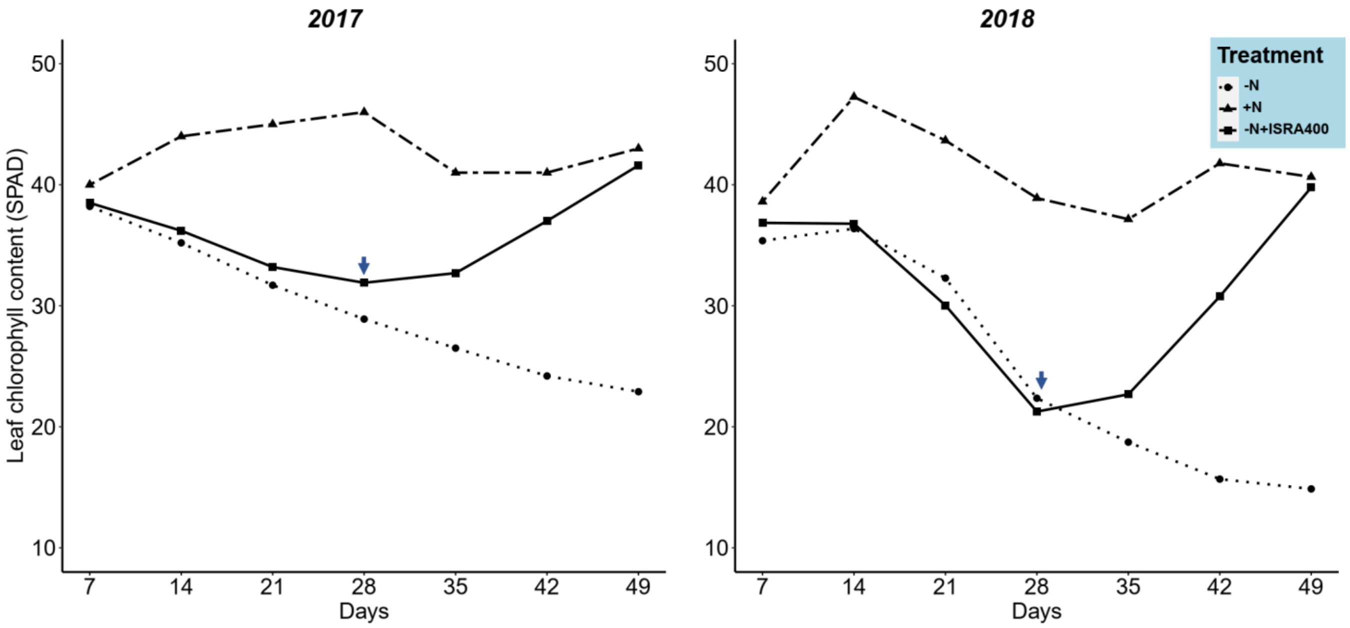

Figure S1. Kinetic of average leaf chlorophyll content in the CSSL population during 49 days after inoculation in shade house experiments. -N and +N treatments are respectively negative (without nitrogen) and positive (with urea) controls. -N+ISRA400 treatment: without nitrogen + inoculation with *Bradyrhizobium vignae* Strain ISRA400. Arrow indicates the start of biological nitrogen fixation.

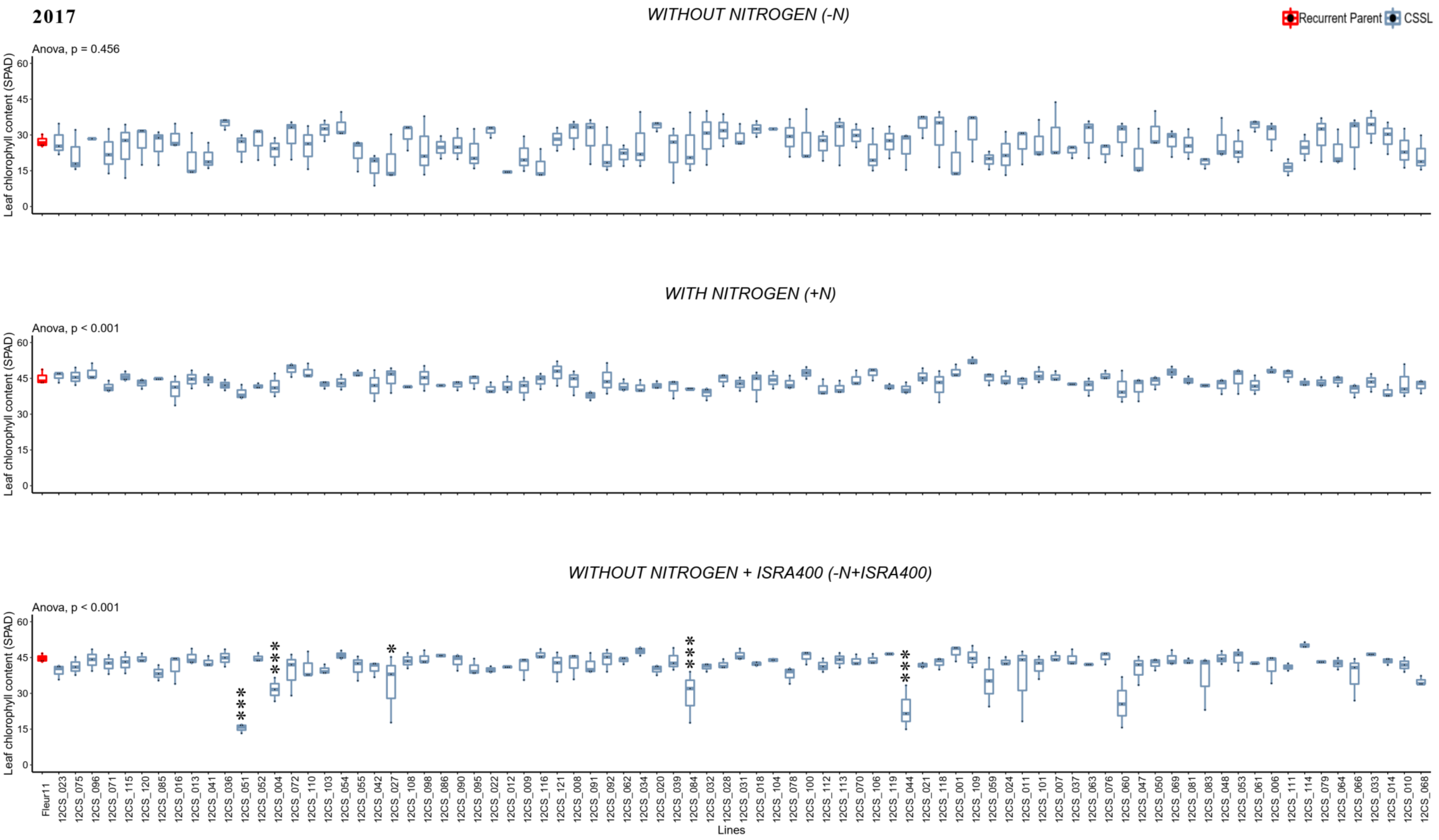

Figure S2. Significant line × leaf chlorophyll content associations observed between Fleur and CSSLs in the 2017 shade house experiment. Without nitrogen and with nitrogen indicate respectively negative and positive controls. -N+ISRA400 treatment: without nitrogen + inoculation with *Bradyrhizobium vignae* Strain ISRA400. *, ** and ***: significant at $p < 0.05$, $p < 0.01$ and $p < 0.001$ respectively using Dunnett multiple comparisons test.

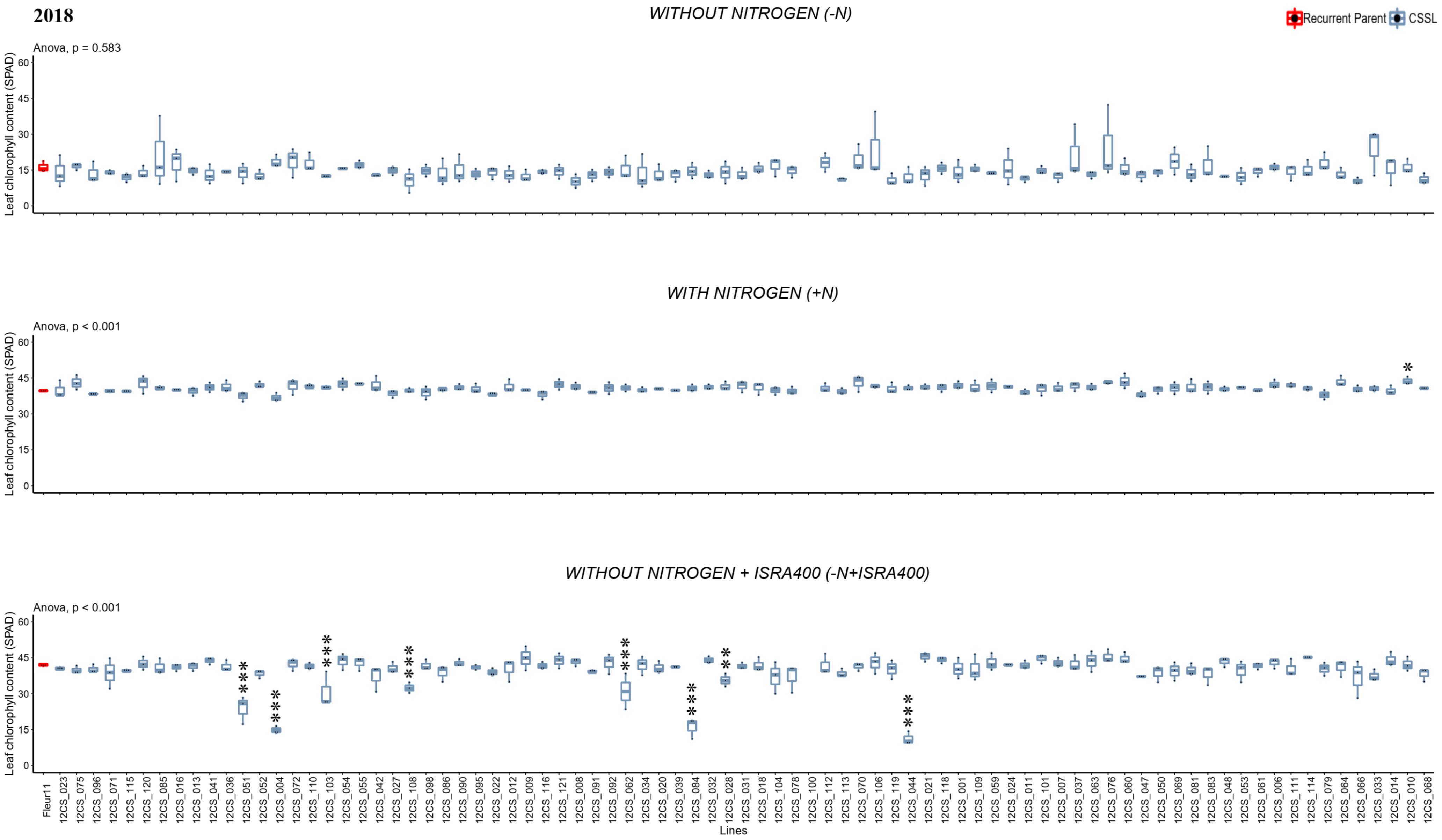

Figure S3. Significant line × leaf chlorophyll content associations observed between Fleur and CSSLs in the 2018 shade house experiment. Without nitrogen and with nitrogen indicate respectively negative and positive controls. -N+ISRA400 treatment: without nitrogen + inoculation with *Bradyrhizobium vignae* Strain ISRA400. *, ** and ***: significant at $p < 0.05$, $p < 0.01$ and $p < 0.001$ respectively using Dunnett multiple comparisons test.

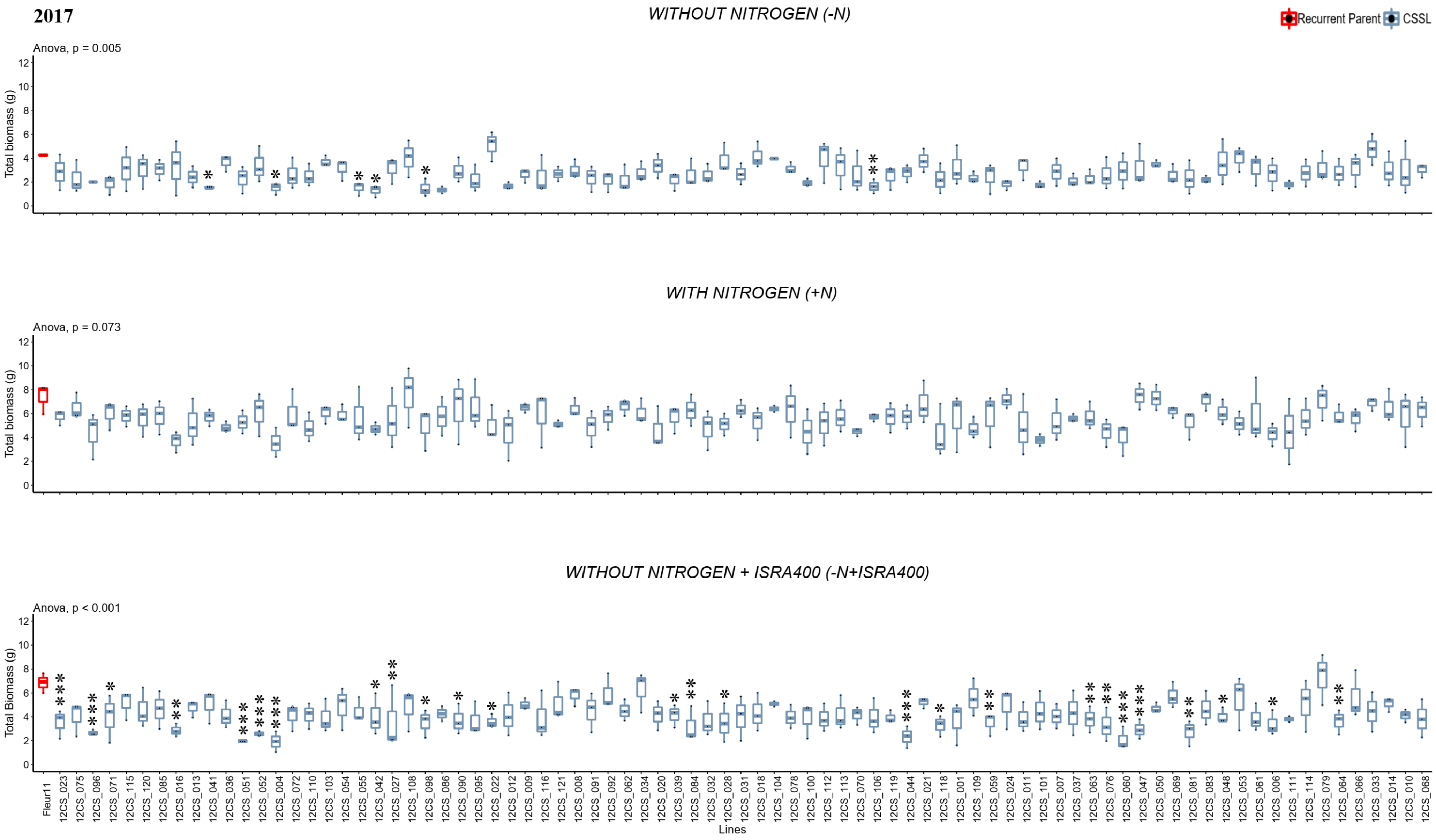

Figure S4. Significant line × total biomass associations observed between Fleur and CSSLs in the 2017 shade house experiment. Without nitrogen and with nitrogen indicate respectively negative and positive controls. -N+ISRA400 treatment: without nitrogen + inoculation with *Bradyrhizobium vignae* Strain ISRA400. *, ** and ***: significant at $p < 0.05$, $p < 0.01$ and $p < 0.001$ respectively using Dunnett multiple comparisons test.

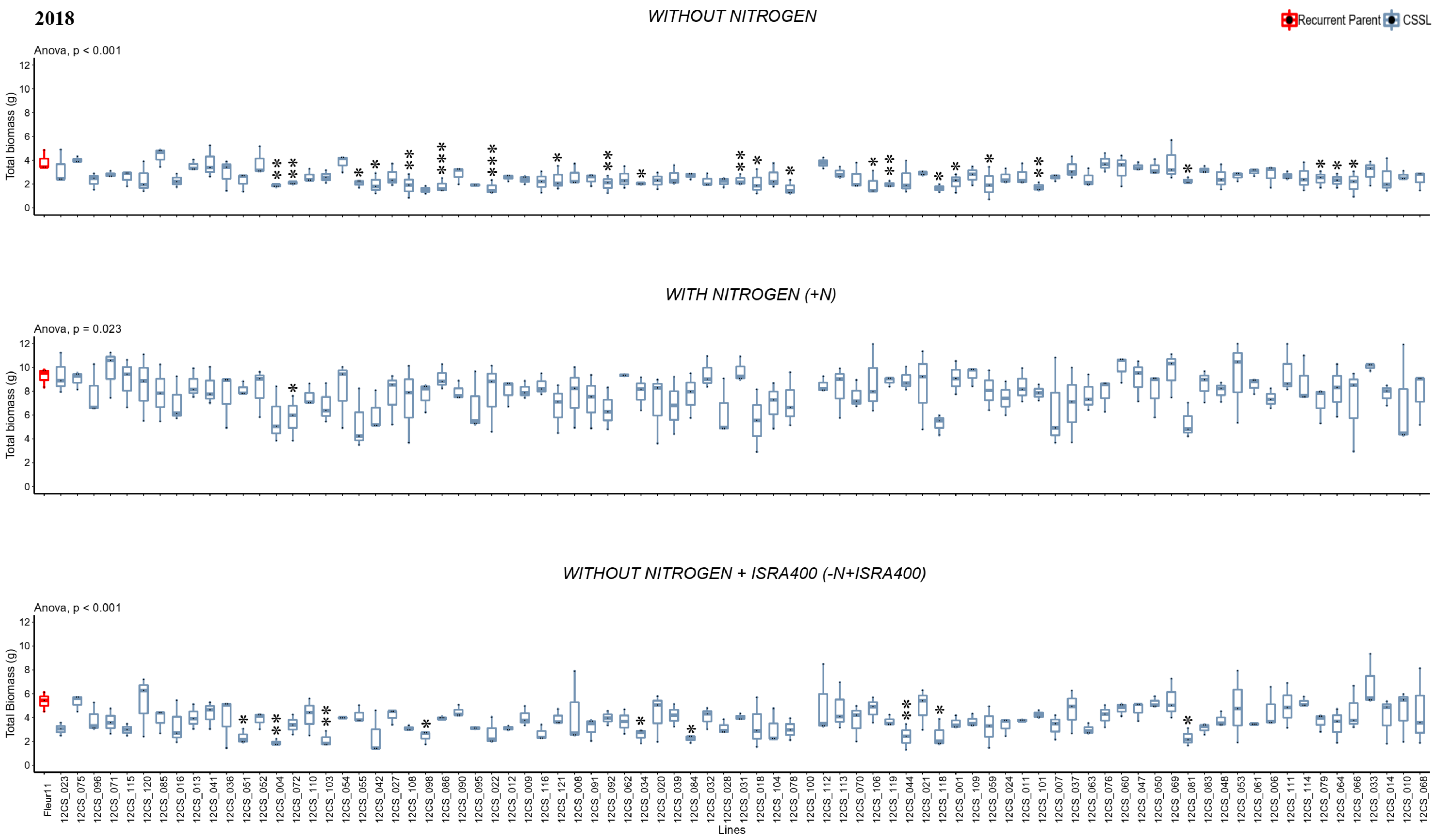

Figure S5. Significant line × total biomass associations observed between Fleur and CSSLs in the 2018 shade house experiment. Without nitrogen and with nitrogen indicate respectively negative and positive controls. -N+ISRA400 treatment: without nitrogen + inoculation with *Bradyrhizobium vignae* Strain ISRA400. *, ** and ***: significant at $p < 0.05$, $p < 0.01$ and $p < 0.001$ respectively using Dunnett multiple comparisons test.
